# Sex-Specific Associations of Triglyceride-Glucose Index and a Body Shape Index with Cardiometabolic Multimorbidity Risk: A Prospective Cohort Study

**DOI:** 10.3390/jcm15114254

**Published:** 2026-05-31

**Authors:** Ting Liu, Yue Li, Yumei Huang, An Pan, Jiajing Yin, Yunfei Liao

**Affiliations:** 1 Department of Geriatrics, Union Hospital, Tongji Medical College, Huazhong University of Science and Technology, Wuhan 430022, China; d202482270@hust.edu.cn; 2 Department of Epidemiology and Biostatistics, Ministry of Education Key Laboratory of Environment and Health, School of Public Health, Tongji Medical College, Huazhong University of Science and Technology, Wuhan 430030, China; 3 Department of Endocrinology, Union Hospital, Tongji Medical College, Huazhong University of Science and Technology, Wuhan 430022, China; 4 Department of Endocrinology and Metabolism, Shanghai Tenth People’s Hospital, School of Medicine, Tongji University, Shanghai 200072, China; 5 Hubei Provincial Clinical Research Center for Diabetes and Metabolic Disorders, Wuhan 430022, China

**Keywords:** CHARLS, insulin resistance, central obesity, risk prediction, prospective cohort study, cardiometabolic multimorbidity

## Abstract

**Background**: Cardiometabolic multimorbidity (CMM) has emerged as a significant global health challenge. The triglyceride-glucose (TyG) index and a body shape index (ABSI), which are markers of insulin resistance and central obesity, respectively, have each been associated with CMM, but their combined utility remains unclear. This study analyzed the association of cumulative and single-point TyG-ABSI with CMM. **Methods**: This study included 5334 participants from the China Health and Retirement Longitudinal Study (CHARLS), covering the period from 2011 to 2020. Cumulative TyG-ABSI was derived from data collected in 2011 and 2015. Incident CMM was defined as having at least two of the following: heart disease, stroke, and diabetes. Cox regression models were used to estimate hazard ratios, and restricted cubic splines (RCS) were employed to examine nonlinear associations. Time-dependent receiver operating characteristic (ROC) curves were constructed to compare predictive performance. Subgroup and sensitivity analyses were also performed. **Results**: Over 9 years of follow-up, 424 participants (7.95%) developed CMM. Those in the highest TyG-ABSI quartile had a 2.46-fold higher risk than those in the lowest quartile. The association varied by sex: cumulative TyG-ABSI showed a nonlinear relationship with a threshold effect in females, whereas in males, only cumulative TyG-ABSI (not the single-point measure) was significant. Cumulative TyG-ABSI demonstrated better 5-year predictive performance than single-point measures. **Conclusions**: Cumulative TyG-ABSI independently predicts CMM risk across sexes, whereas single-point TyG-ABSI is only predictive in females. Quantifying long-term metabolic burden using cumulative TyG-ABSI offers a practical, noninvasive tool for chronic risk stratification in aging populations. Routine assessment of cumulative TyG-ABSI in clinical and community settings could facilitate early identification of high-risk individuals, enabling targeted preventive interventions to reduce the growing burden of CMM.

## 1. Introduction

Cardiometabolic multimorbidity (CMM), defined as the coexistence of at least two cardiometabolic conditions such as heart disease, stroke, and diabetes, has become a major global health concern, driven largely by population aging and the obesity epidemic [1,2]. CMM is associated with elevated mortality, cognitive decline, and reduced quality of life [3,4,5].

The combined use of metabolic and anthropometric indices has demonstrated superior ability in identifying individuals at increased cardiometabolic risk compared with single-domain markers [6,7], as these integrated metrics capture the multifactorial complexity of metabolic syndrome more comprehensively [8]. The triglyceride-glucose (TyG) index reliably captures insulin resistance (IR) [9,10], while a body shape index (ABSI) independently reflects visceral adiposity, as validated by bioelectrical impedance and MRI [11,12]. Although TyG-WHtR, TyG-WC, and TyG-BMI have shown predictive efficacy for cardiometabolic outcomes [13,14], they predominantly reflect general adiposity rather than the visceral-IR axis. The TyG-ABSI bridges this gap by capturing the bidirectional amplification between visceral adiposity and insulin resistance: excess visceral adipose tissue releases substantial free fatty acids and cytokines into the portal vein that alter hepatic metabolism and promote an insulin-resistant state upon delivery to the liver [15]; conversely, insulin resistance engenders a lipotoxic and inflammatory milieu that aggravates visceral adiposity and disrupts insulin signaling through shared pathophysiological pathways [16]. Consequently, this composite index better represents the integrated metabolic burden than either indicator alone.

Nevertheless, prior evidence on TyG-ABSI relies predominantly on single-time-point measurements [17,18,19,20], which are vulnerable to transient fluctuations and fail to reflect the cumulative metabolic burden accrued over years of exposure. Long-term cumulative metabolic burden integrates both magnitude and duration of metabolic insult, thereby more faithfully reflecting the chronic pathophysiological processes and progressive multisystem decompensation observed in aging populations than do isolated snapshots. This paradigm is further shaped by sex-specific life-course determinants: women experience distinct metabolic trajectories influenced by reproductive history and adiposity redistribution across the menopausal transition, rendering central adiposity and IR particularly relevant to postmenopausal cardiometabolic risk [21].

Accordingly, this study examines whether cumulative TyG-ABSI exposure predicts incident CMM more strongly than single-point TyG-ABSI, characterizes sex-specific dose–response patterns and potential metabolic thresholds, and evaluates its incremental predictive utility.

## 2. Methods

### 2.1. Data Source and Study Population

A prospective cohort study was carried out using data from CHARLS, which is a nationally representative longitudinal cohort comprising middle-aged and older individuals. The initial survey enrolling 17,708 participants who were 45 years old or older, sourced from 450 communities across 150 counties in 28 provinces. Follow-up surveys were subsequently conducted during 2013–2014, 2015–2016, 2017–2018, and 2019–2020. The study used publicly available data for secondary analysis (original data collection approved under IRB00001052-11015). Ethical approval was not required for this secondary analysis, and informed consent was waived [22]. The data are publicly accessible via the CHARLS website (http://charls.pku.edu.cn, accessed on 27 July 2025).

**Figure 1:**
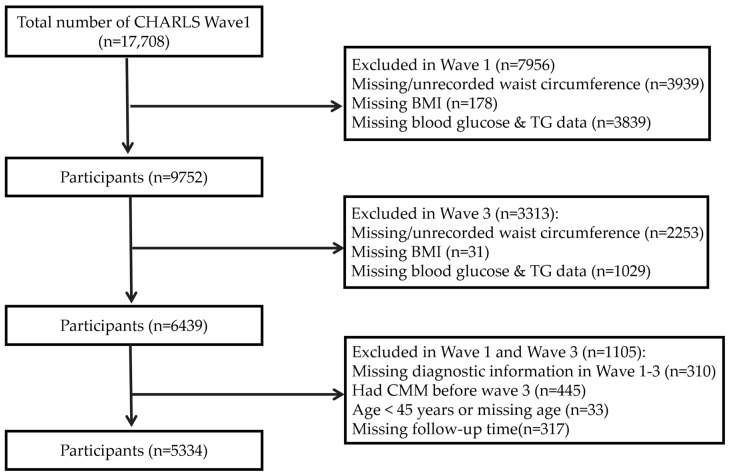
Flow diagram of participant enrollment and exclusion.

From the 17,708 individuals who participated in CHARLS waves from 2011 to 2020, we applied the following sequential inclusion and exclusion criteria: (1) missing data for TyG-ABSI calculation at Wave 1 (*n* = 7956); (2) missing data at Wave 3 (*n* = 3313); (3) incomplete CMM diagnostic information (*n* = 310); (4) pre-existing CMM before Wave 3 (*n* = 445); (5) age < 45 years or missing age (*n* = 33); and (6) missing follow-up time (*n* = 317). The final analytical sample comprised 5334 participants (Figure 1). Excluded participants were younger, more likely to be male, less likely to be rural residents, had lower educational attainment, and had higher prevalences of diabetes, stroke, and heart disease compared with the included sample (Supplementary Table S5). Thus, our findings are most generalizable to older, female, rural, and less educated populations with lower baseline cardiometabolic burden.

Assessment of TyG-ABSI

Single-point TyG-ABSI was calculated using data from Wave 3 [23,24]:

$$\mathrm{ABSI}=WC÷\left({BMI}^{2/3}\times {Height}^{1/2}\right)$$

TyG index = In [fasting triglyceride (mg/dL) × fasting glucose (mg/dL)/2]

single-point TyG-ABSI = TyG index × ABSI

Cumulative TyG-ABSI was derived from Wave 1 and Wave 3, using the trapezoidal area-under-the-curve (AUC) method [25]:

Cumulative TyG-ABSI = (TyG-ABSI_2011_ + TyG-ABSI_2015_)/2 × (2015 − 2011)

Due to the availability of biochemical measurements in CHARLS, cumulative TyG-ABSI was calculated using only two time points. This approach assumes an approximately linear metabolic trajectory between assessments and may not capture non-monotonic fluctuations. Consequently, the resulting metric represents an estimated integrated exposure rather than a precisely measured cumulative dose, and future studies with more frequent repeated measures are warranted to validate these findings.

### 2.2. Definition of CMM

The primary outcome was incident CMM which is defined as the presence of a minimum of two cardiometabolic conditions, such as stroke, diabetes, and heart disease [26]. The diagnostic criteria were as follows:

Heart disease: Participants were considered affected if they reported a physician diagnosis of heart disease. It is important to note that the heart disease variable in CHARLS aggregates atherosclerotic conditions with potentially non-atherosclerotic conditions (e.g., congestive heart failure, valvular disease, arrhythmias). This heterogeneity may introduce outcome misclassification because the metabolic drivers differ across these phenotypes. Nevertheless, this definition is dictated by the original CHARLS data structure—participants were asked a single question regarding physician-diagnosed heart disease without further subtyping—and has been consistently applied in previous CHARLS-based studies [27,28]. We therefore interpret our findings as reflecting the association with the composite cardiometabolic endpoint as operationalized in this cohort, rather than specific pathophysiological subtypes.

Stroke: Participants were considered affected if they reported a physician diagnosis of stroke. These data were collected through standardized questionnaires at each wave.

Diabetes was diagnosed when any of the following criteria were met: (1) a physician’s diagnosis of diabetes or hyperglycemia reported by the individual; (2) current use of glucose-lowering drugs; (3) FBG of 126 mg/dL or higher; or (4) glycated hemoglobin (HbA1c) levels at or above 6.5%.

### 2.3. Covariate Assessment

Drawing upon the existing literature and clinical expertise, this study incorporated five categories of covariates: demographic characteristics (age, sex, educational level, place of residence and marital status); physical measurements (systolic blood pressure [SBP] and diastolic blood pressure [DBP]); lifestyle factors (current smoking and current alcohol use); laboratory parameters (serum creatinine [Scr], blood urea nitrogen [BUN], low-density lipoprotein cholesterol [LDL-C], high-density lipoprotein cholesterol [HDL-C], and triglycerides [TG]); and cancer history (presence or absence). Blood samples were obtained after fasting, and analytical methods were aligned with those employed in previous studies.

### 2.4. Missing Data Handling and Data Preprocessing

Missing data were handled using multiple imputation by chained equations (MICE) with the mice package in R (version 4.5.1). Five complete datasets were generated. The variables included in the imputation model are detailed in the Supplementary Methods. Covariates were selected for inclusion based on established epidemiological evidence and clinical plausibility. The fully adjusted model (Model 3) included demographic characteristics (age, sex, educational attainment, marital status, residence), lifestyle factors (current smoking, current alcohol consumption), metabolic parameters (SBP, DBP, TG, HDL-C, LDL-C), kidney function markers (Scr, BUN), and history of cancer. Multicollinearity was assessed using the generalized variance inflation factor (GVIF); no significant collinearity was detected (Supplementary Table S10.2). To mitigate the effects of outliers, the TyG and ABSI indices were Winsorized at the extreme percentiles; details are provided in the Supplementary Methods.

### 2.5. Statistical Analysis

Continuous variables are reported as the mean ± standard deviation (SD) when the data follow a normal distribution, or as the median along with the interquartile range (IQR) for distributions that are skewed. Categorical variables are shown as frequencies and percentages. The relationship between TyG-ABSI and CMM risk was examined using multivariable Cox proportional hazards regression. The proportional hazards assumption was formally tested using Schoenfeld residual-based tests. No significant violations were observed for the primary exposures (cumulative TyG-ABSI: χ^2^ = 0.235, *p* = 0.628; single-point TyG-ABSI: χ^2^ = 0.174, *p* = 0.677) or the global test (cumulative model: χ^2^ = 24.6, df = 18, *p* = 0.135; single-point model: χ^2^ = 25.1, df = 18, *p* = 0.122). A marginally significant time-dependent effect was observed for LDL-C (*p* = 0.033 and *p* = 0.032, respectively); however, given the non-significant global test and the absence of meaningful effect modification, this single covariate deviation at the conventional threshold is unlikely to materially influence the robustness of our primary conclusions (Supplementary Table S11). The results are presented as hazard ratios (HRs) with 95% confidence intervals (CIs). We constructed three progressively adjusted Cox models: Model 1 was unadjusted; Model 2 was adjusted for age and sex; and Model 3 was further adjusted for history of cancer, marital status, smoking status, drinking status, educational level, residence, BUN, Scr, TG, HDL-C, LDL-C, SBP, and DBP. To explore dose–response relationships, restricted cubic spline (RCS) regression analyses with four knots were performed to assess nonlinearity. The threshold effect was examined using a two-stage Cox regression model, with optimal thresholds determined through log-likelihood ratio tests. Because the reference group in the female subgroup had a small number of events (<10), the Firth penalized likelihood method was used to reduce sparse-data bias. Time-dependent receiver operating characteristic (ROC) curves were constructed to evaluate 5-year predictive performance. To compare predictive value, integrated discrimination improvement (IDI) and net reclassification improvement (NRI) were calculated. Subgroup analyses stratified by sex, marital status, smoking status, and other characteristics were performed as exploratory analyses. Sensitivity analyses included complete-case analysis, exclusion of individuals with a history of stroke, heart disease, or diabetes at Wave 3, and E-value calculation. In addition, participants with a history of cancer were excluded to address potential confounding from metabolic disorders caused by malignant tumors.

All statistical analyses were performed using EmpowerStats (version 5.3) and R software (version 4.5.1). All tests were two-sided, and a *p*-value < 0.05 was considered statistically significant.

## 3. Results

### 3.1. Characteristics of the Study Population

Over a median follow-up of 9.0 years, 424 incident CMM cases occurred among 5334 participants. The mean age was 62.2 ± 8.7 years, and 55.3% were female. The mean cumulative TyG-ABSI was 2.13 ± 0.25. Participants with higher cumulative TyG-ABSI (Q4 vs. Q1) tended to be older, more frequently female, less educated, unmarried, and urban residents (all *p* < 0.05). They also had higher BMI, blood pressure, triglycerides, and HbA1c, and lower HDL-C (all *p* < 0.001). The prevalence of hypertension and diabetes increased across quartiles (both *p* < 0.001), whereas the prevalence of heart disease and stroke did not differ significantly (both *p* > 0.05) (Table 1). Baseline characteristics stratified by single-point TyG-ABSI quartiles are presented in Table S1.

### 3.2. Association of Cumulative and Single-Point TyG-ABSI with Incident CMM

In multivariable Cox proportional hazards models, cumulative TyG-ABSI was associated with a higher risk of incident CMM across all three models (Table 2). In Model 3, each 1-SD increase in cumulative TyG-ABSI was associated with a 39% higher risk of CMM (HR 1.39, 95% CI 1.21–1.59, *p* < 0.001). Participants in the highest quartile (Q4) had a 2.46-fold higher risk compared with those in the lowest quartile (Q1) (HR 2.46, 95% CI 1.74–3.49, *p* < 0.001), with a significant dose–response trend across quartiles (*p* for trend < 0.001).

Single-point TyG-ABSI was also positively associated with CMM risk, although the effect size was smaller. In Model 3, each 1-SD increase was associated with a 33% higher risk (HR 1.33, 95% CI 1.13–1.56, *p* < 0.001), and participants in Q4 had a twofold higher risk compared with Q1 (HR 2.00, 95% CI 1.40–2.85, *p* < 0.001).

### 3.3. Dose–Response and Threshold Analyses

**Figure 2:**
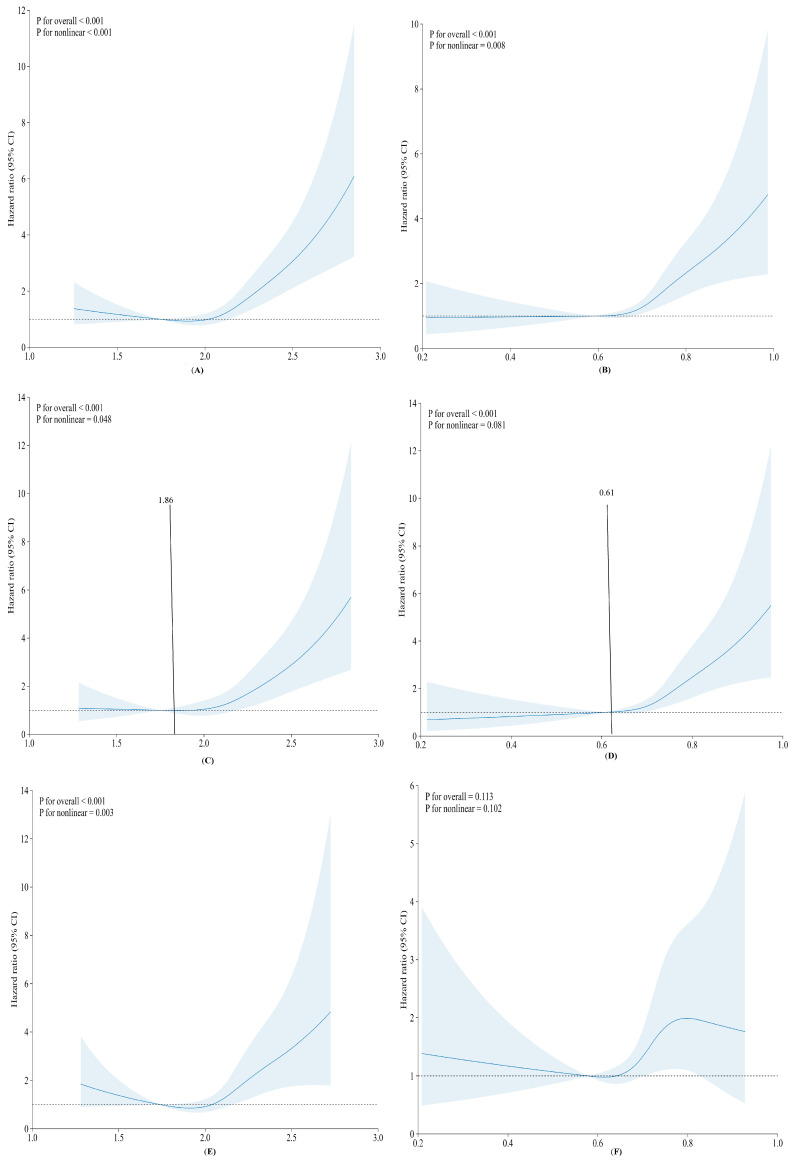
Dose–response relationships between single-point and cumulative TyG-ABSI and CMM incidence using restricted cubic spline models. (**A**) Cumulative TyG-ABSI in the total population; (**B**) single-point TyG-ABSI in the total population; (**C**) cumulative TyG-ABSI in females; (**D**) single-point TyG-ABSI in females; (**E**) cumulative TyG-ABSI in males; (**F**) single-point TyG-ABSI in males. The blue solid line represents the fitted hazard ratio (HR), the light blue shaded area represents the 95% confidence interval (CI), and the black dashed line represents the reference line at HR = 1. Model 3 was adjusted for age, sex (for total population analyses), smoking status, serum creatinine, marital status, educational level, residence, drinking status, history of cancer, TG, SBP, DBP, blood urea nitrogen, HDL, and LDL.

RCS analyses revealed significant nonlinear relationships (Figure 2). In females, cumulative TyG-ABSI exhibited a nonlinear pattern (*p* for nonlinearity = 0.048) with a threshold effect: an inflection point at 1.86, above which the risk increased sharply (HR 7.13; *p* < 0.001). Single-point TyG-ABSI also showed a near-linear association in females (threshold at 0.61; HR 2.43; *p* = 0.032). In males, only cumulative TyG-ABSI was significantly associated with CMM (*p* for overall < 0.001), and the relationship was nonlinear (*p* for nonlinearity = 0.003); single-point TyG-ABSI was not significant (*p* for overall = 0.113). Detailed threshold results are provided in Table S6.

### 3.4. Predictive Performance

**Figure 3:**
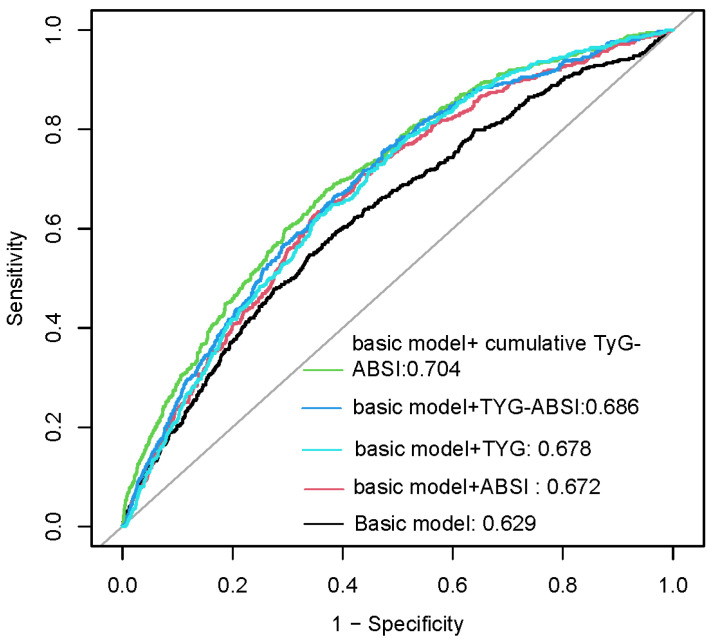
Comparison of time-dependent ROC curves for different models predicting 5-year CMM risk. The colored curves represent the basic model and its sequential additions of ABSI, TyG, single-point TyG-ABSI, and cumulative TyG-ABSI, with corresponding AUC values shown in the legend. The grey diagonal line represents the reference line of no discrimination (AUC = 0.5), indicating random guessing. TyG, triglyceride-glucose; ABSI, a body shape index; TyG-ABSI, glucose triglyceride-a body shape index.

Time-dependent ROC analysis showed that the base model had an AUC of 0.629 (95% CI 0.601–0.657) for 5-year CMM risk (Figure 3). Sequential addition of ABSI, TyG, single-point TyG-ABSI, and cumulative TyG-ABSI progressively improved discriminative ability, with AUC values of 0.672, 0.678, 0.686, and 0.704, respectively. The addition of cumulative TyG-ABSI yielded the largest improvement in reclassification and discrimination, with an IDI of 0.032 and an NRI of 0.101 (Table S7).

### 3.5. Subgroup Analyses

**Figure 4:**
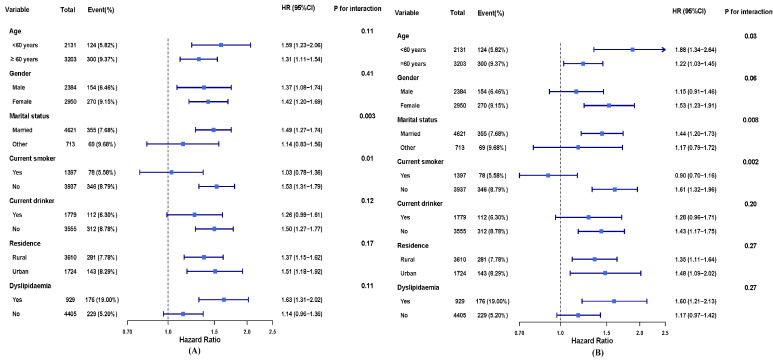
Forest plots of subgroup analyses for the association between single-point and cumulative TyG-ABSI and CMM risk. (**A**) cumulative TyG-WHtR (**B**) TyG-ABSI.

In exploratory subgroup analyses, the associations of cumulative and single-point TyG-ABSI with CMM were generally consistent across most strata (Figure 4). However, marital status and smoking status appeared to modify the association of single-point TyG-ABSI with CMM, with stronger associations observed among married individuals and non-smokers. These subgroup differences should be interpreted cautiously, as they may reflect residual confounding, selection bias, or limited statistical power within subgroups rather than true biological effect modification.

Sex-stratified analyses showed that single-point TyG-ABSI was significantly associated with CMM in women (HR 1.53, 95% CI 1.23–1.91) but not in men (HR 1.15, 95% CI 0.91–1.46). By contrast, cumulative TyG-ABSI was significantly associated with CMM in both sexes.

### 3.6. Sensitivity Analyses

The main findings remained robust across multiple sensitivity analyses. Results were consistent in complete-case analyses and across the five imputed datasets (Table S2). Exclusion of participants with stroke, heart disease, or diabetes at Wave 3 did not materially alter the associations (Table S3). Further exclusion of participants with a history of cancer also yielded similar results (Table S4). E-value analyses indicated that an unmeasured confounder would need to be associated with both the exposure and the outcome by a risk ratio of at least 1.67 to fully explain away the observed associations (Table S8).

## 4. Discussion

Recent studies have established baseline associations between TyG-ABSI and individual cardiometabolic outcomes. However, the longitudinal cumulative exposure to TyG-ABSI and its sex-specific nonlinear relationship with CMM remain inadequately elucidated. This study expands upon existing evidence by demonstrating that cumulative TyG-ABSI is more effective than single-point measures and identifies distinct metabolic thresholds (1.86 for females) for risk stratification.

Previous research on TyG-ABSI has predominantly focused on individual cardiometabolic diseases or mortality endpoints [29,30]. Our findings corroborate this literature by confirming a significant nonlinear dose–response relationship [31,32], yet the effect size for CMM appears relatively pronounced, suggesting that the synergy between insulin resistance and central obesity captured by TyG-ABSI may exert an amplified effect on multisystem injury.

The observed association may be partially explained by the synergistic amplification of insulin resistance and central adiposity. Potential mechanisms include hyperglycemia-driven endothelial dysfunction [15] concurrent with visceral adipose tissue releasing cytokines, including pro-inflammatory mediators such as interleukin-6, into the portal circulation [16,33]. With advancing age, chronic metabolic dysfunction in older adults may trigger systemic damage through low-grade inflammation, ectopic fat deposition, and altered body composition, thereby creating a self-reinforcing vicious cycle that increases vulnerability to cardiovascular and metabolic disorders [34,35,36,37,38]. While this framework is biologically plausible, these pathways remain hypothetical in the context of our observational design and require experimental validation.

Our finding of a threshold effect in women (cumulative TyG-ABSI > 1.86) suggests a biological tipping point where compensatory mechanisms are overwhelmed, leading to lipotoxicity, mitochondrial dysfunction, and multi-organ damage [39,40]. Premenopausal women generally exhibit preferential subcutaneous fat storage and higher insulin sensitivity than men [41]. Following menopause, declining estrogen levels are associated with visceral fat accumulation and reduced subcutaneous adipose expandability, as adipocyte hypertrophy, inflammation, and fibrosis progressively limit lipid-buffering capacity [42,43]. This pattern is consistent with the adipose tissue expandability hypothesis and may account for the steep increase in CMM risk observed beyond a specific metabolic threshold in our female participants [44]. These postulated mechanisms are speculative and warrant dedicated experimental and longitudinal hormonal studies to confirm.

Subgroup analyses revealed that in women, both single-point and cumulative TyG-ABSI were significantly associated with CMM, whereas in men, only the cumulative metric achieved significance. Single-point assessments capture transient metabolic fluctuations that may be more pronounced in men, whereas cumulative exposure integrates both the duration and intensity of dysregulation. Biologically, sex hormones regulate insulin sensitivity and body composition in a sex-specific manner. In aging men, testosterone deficiency is associated with reduced muscle mass and increased visceral adiposity, as low testosterone decreases lipoprotein lipase activity and impairs lipolytic signaling in adipocytes [45,46]. In postmenopausal women, declining estrogen levels reduce subcutaneous fat storage capacity and promote visceral fat redistribution, thereby increasing susceptibility to hepatic steatosis [47,48]. These divergent trajectories may explain why cumulative TyG-ABSI is required to capture men’s sustained metabolic risk, while women exhibit significant associations with both measures. Nevertheless, these interpretations are tentative and should be treated as hypothesis-generating. Exploratory subgroup analyses indicated stronger associations among married individuals and non-smokers, but these findings were not prespecified and should be interpreted with caution.

Cumulative TyG-ABSI was associated with statistically significant improvement in discrimination (AUC increase from 0.629 to 0.704). Nevertheless, this incremental predictive value should be interpreted cautiously, as AUC alone may not capture clinical utility, and the moderate discrimination suggests substantial overlap between risk groups. External validation in independent cohorts is essential before clinical implementation.

This study possesses several strengths. First, we examined CMM as a composite outcome reflecting multi-system dysfunction, which offers greater clinical relevance than single-disease endpoints for aging populations. Second, we applied sex-stratified restricted cubic spline models and threshold effect analyses. Third, we used Firth penalized likelihood to address sparse-data bias in the female subgroup, a methodological refinement not previously reported in TyG-index studies.

Nonetheless, several limitations must be acknowledged. First and foremost, the observational design precludes causal inference; the observed associations should be interpreted as statistical relationships rather than evidence of direct causation. Residual confounding from unmeasured variables may have influenced the results. Furthermore, disease status was ascertained based on self-reported physician diagnoses—an approach that carries inherent misclassification risk and may bias effect estimates toward the null [49]. Second, the definition of heart disease in CHARLS aggregates atherosclerotic conditions with potentially non-atherosclerotic conditions (congestive heart failure, valvular disease, arrhythmias, or cardiomyopathies). This heterogeneity may introduce noise and limit the precision of our risk estimates, as not all included conditions share identical pathophysiological pathways. Third, TyG-ABSI partially overlaps with CMM diagnostic criteria (both incorporate FBG and TG), which may inflate associations despite multivariate adjustment. Fourth, participants excluded due to missing data were younger and had a higher cardiometabolic burden, suggesting that our findings may be most applicable to older individuals with more complete health records. Finally, cumulative TyG-ABSI was calculated using only two time points (2011 and 2015), which may not fully capture dynamic longitudinal trajectories; future studies with more frequent repeated measures are warranted. In the female subgroup, the reference group for single-point TyG-ABSI had a low event rate. We applied Firth penalized likelihood to mitigate sparse-data bias; although results support a threshold effect in women, the precise magnitude should be interpreted with caution and validated in larger cohorts.

## 5. Conclusions

This study indicates that cumulative TyG-ABSI is an independent indicator of CMM risk, surpassing single-point measurements and traditional metabolic indices. By simultaneously reflecting chronic insulin resistance and central adiposity, it holds promise for risk stratification. However, external validation in independent populations is warranted before cumulative TyG-ABSI can be recommended as a practical screening tool for early identification of high-risk individuals and for guiding timely, targeted interventions.

## Figures and Tables

**Table 1:** Baseline characteristics of participants by cumulative TyG-ABSI Quartile.

Characteristic	Total N = 5334	Q1 N = 1334	Q2 N = 1333	Q3 N = 1333	Q4 N = 1334	*p*-Value
Age, years (Mean ± SD)	62.24 ± 8.70	60.77 ± 8.59	61.73 ± 8.53	62.23 ± 8.66	64.23 ± 8.65	<0.001
Sex, *n* (%)						<0.001
Female	2950 (55.31%)	611 (45.80%)	696 (52.21%)	781 (58.59%)	862 (64.62%)	
Male	2384 (44.69%)	723 (54.20%)	637 (47.79%)	552 (41.41%)	472 (35.38%)	
Education, *n* (%)						<0.001
High school or above	479 (8.98%)	127 (9.52%)	132 (9.90%)	129 (9.68%)	91 (6.82%)	
Middle school	1099 (20.60%)	299 (22.41%)	287 (21.53%)	281 (21.08%)	232 (17.39%)	
Primary school	1220 (22.87%)	292 (21.89%)	323 (24.23%)	308 (23.11%)	297 (22.26%)	
No formal education	2536 (47.54%)	616 (46.18%)	591 (44.34%)	615 (46.14%)	714 (53.52%)	
Marriage, *n* (%)						0.008
Married	4621 (86.63%)	1174 (88.01%)	1176 (88.22%)	1162 (87.17%)	1109 (83.13%)	
Other	713 (13.37%)	160 (11.99%)	157 (11.78%)	171 (12.83%)	225 (16.87%)	
Residence, *n* (%)						<0.001
Rural	3610 (67.68%)	967 (72.49%)	930 (69.77%)	876 (65.72%)	837 (62.74%)	
Urban	1724 (32.32%)	367 (27.51%)	403 (30.23%)	457 (34.28%)	497 (37.26%)	
Hypertension, *n* (%)	2753 (51.61%)	542 (40.94%)	627 (47.32%)	729 (55.10%)	855 (64.58%)	<0.001
Diabetes, *n* (%)	366 (6.86%)	100 (7.50%)	105 (7.88%)	177 (13.28%)	394 (29.54%)	<0.001
Heart disease, *n* (%)	693 (12.99%)	155 (11.62%)	186 (13.95%)	194 (14.55%)	158 (11.84%)	0.052
Current smoker, *n* (%)	1398 (26.21%)	424 (31.78%)	371 (27.85%)	308 (23.14%)	294 (22.04%)	<0.001
Current Drinker, *n* (%)	1778 (33.33%)	531 (39.81%)	450 (33.76%)	414 (31.08%)	382 (28.66%)	<0.001
BMI, mmHg (Mean ± SD)	23.77 ± 3.62	22.77 ± 3.49	23.42 ± 3.55	24.18 ± 3.66	24.72 ± 3.46	<0.001
SBP, mmHg (Mean ± SD)	127.75 ± 19.92	123.24 ± 19.03	126.24 ± 19.68	128.50 ± 19.31	132.90 ± 20.38	<0.001
DBP, Mean (SD)	75.01 ± 11.73	73.23 ± 11.47	74.74 ± 11.87	75.47 ± 11.58	76.62 ± 11.69	<0.001
HDL, mg/dL (Mean ± SD)	51.72 ± 11.87	55.61 ± 13.22	53.46 ± 11.79	50.64 ± 10.67	47.17 ± 9.82	<0.001
LDL, mg/dL (Mean ± SD)	103.33 ± 28.96	98.38 ± 26.45	104.17 ± 28.14	107.47 ± 28.86	103.29 ± 31.45	<0.001
TG, mg/dL median (IQR)	115.04 (83.19–168.14)	80.53 (64.60–104.42)	97.35 (76.99–125.66)	130.97 (99.12–174.34)	189.38 (139.82–275.66)	<0.001
Uric acid, mg/dL (Mean ± SD)	4.88 ± 1.39	4.63 ± 1.29	4.76 ± 1.36	4.96 ± 1.34	5.17 ± 1.48	<0.001
HbA1c, (Mean ± SD)	5.96 ± 0.94	5.74 ± 0.54	5.77 ± 0.49	5.91 ± 0.74	6.42 ± 1.48	<0.001
Stroke, *n* (%)	96 (1.80%)	19 (1.42%)	29 (2.18%)	24 (1.80%)	24 (1.80%)	0.546
CMM, *n* (%)	424 (7.95%)	57 (4.27%)	83 (6.23%)	106 (7.95%)	178 (13.34%)	<0.001

CMM: cardiometabolic multimorbidity; BMI: body mass index; TG: triglyceride; HDL: high-density lipoprotein cholesterol; SBP: Systolic blood pressure; HbA1c: glycated haemoglobin; LDL: low-density lipoprotein cholesterol; DBP: diastolic blood pressure; SD: standard deviation.

**Table 2:** Association between Cumulative/single-point TyG-ABSI and CMM.

	Model 1		Model 2		Model 3	
	HR (95% CI)	*p* Value	HR (95% CI)	*p* Value	HR (95% CI)	*p* Value
single-point TyG-ABSI (per 1 SD)	1.51 (1.34, 1.70)	<0.001	1.43 (1.26, 1.61)	<0.001	1.33 (1.13, 1.56)	<0.001

1	1 (Ref)		1 (Ref)		1 (Ref)	
2	1.17 (0.84, 1.63)	0.347	1.14 (0.82, 1.59)	0.436	1.05 (0.75, 1.47)	0.768
3	1.85 (1.37, 2.50)	<0.001	1.76 (1.29, 2.38)	<0.001	1.52 (1.11, 2.10)	0.013
4	2.59 (1.94, 3.45)	<0.001	2.32 (1.73, 3.10)	<0.001	2.00 (1.40, 2.85)	<0.001
Trend.test		<0.001		<0.001		<0.001
Cumulative TyG-ABSI (per 1 SD)	1.53 (1.38, 1.69)	<0.001	1.45 (1.31, 1.61)	<0.001	1.39 (1.21, 1.59)	<0.001
1	1 (Ref)		1 (Ref)		1 (Ref)	
2	1.45 (1.04, 2.04)	0.028	1.40 (1.00, 1.97)	0.049	1.29 (0.92, 1.82)	0.144
3	1.88 (1.36, 2.59)	<0.001	1.76 (1.27, 2.43)	<0.001	1.56 (1.11, 2.17)	0.01
4	3.21 (2.38, 4.32)	<0.001	2.85 (2.10, 3.86)	<0.001	2.46 (1.74, 3.49)	<0.001
Trend.test		<0.001		<0.001		<0.001

Model 1 was unadjusted. Model 2 adjusted for age, sex. Model 3 adjusted for age, sex, history of cancer, marital status, smoking status, drinking status, educational level, residence, BUN, Scr, TG, HDL-C, LDL-C, SBP and DBP. Ref: reference; CI: confidence interval; HR: hazard ratio.

## Data Availability

The datasets generated and analyzed during the current study are available in the China Health and Retirement Longitudinal Study (CHARLS) repository, [http://charls.pku.edu.cn].

## Supplementary Materials

The following supporting information can be downloaded at: https://www.mdpi.com/article/10.3390/jcm15114254/s1, Table S1: Characteristics of the study population according to TYG-ABSI at Wave 3; Table S2: Cox regression analysis for the association between TyG-ABSI and CMM (using the original data analysis); Table S3: Sensitivity analysis of the TyG-ABSI on the risks of developing CMM in participants without diabetes, heart disease or stroke (N = 3720); Table S4: Sensitivity analysis of the TyG-ABSI on the risks of developing CMM in participants without diabetes, heart disease, stroke and cancer (N = 3679); Table S5: Comparison of characteristics between excluded and included populations; Table S6: Threshold effect analysis of TyG-ABSI on CMM risk in females; Table S7: Comparison of the predictive performance for CMM; Table S8: Associations of single-point and cumulative TyG-ABSI with incident CMM and E-value analysis; Table S9: Sensitivity analysis of the TyG-ABSI on the risks of developing CMM; Table S10.1: Covariance analysis of cumulative TyG-ABSI and other variables based on model 3; Table S10.2: Covariance analysis of TyG-ABSI and other variables based on model 3; Table S11: Results of Schoenfeld residual-based tests for proportional hazards assumption in cumulative and single-point TyG-ABSI models.

## Abbreviations

CMM	Cardiometabolic multimorbidity
CHARLS	Chinese health and retirement longitudinal study
HR	Hazard ratio
CI	Confidence interval
SD	Standard deviation
IQR	Interquartile range
AUC	Area under the curve
IR	Insulin resistance
TYG	Triglyceride-glucose index
ABSI	A Body Shape Index
FPG	Fasting plasma glucose
RCS	Restricted cubic spline
Scr	Serum creatinine
WC	Waist circumference
HbA1c	Hemoglobin A1c
ROC	Receiver operating characteristic
BMI	body mass index
SBP	Systolic blood pressure
BUN	Blood urea nitrogen
DBP	Diastolic blood pressure
TG	Triglyceride
HDL-C	High density lipoprotein cholesterol
LDL-C	Low density lipoprotein cholesterol
