# Supplementary material for: Sex-Specific Associations of Triglyceride-Glucose Index and a Body Shape Index with Cardiometabolic Multimorbidity Risk: A Prospective Cohort Study
Source: J Clin Med. 2026 May 31;15(11):4254. doi: 10.3390/jcm15114254 (PMC13257997; doi:10.3390/jcm15114254)
Supplement: Supplementary file 1 [file jcm-15-04254-s001.zip › jcm-4314552-supplementary.pdf]

Supplementary Methods

Covariate definitions

The following covariates were used in the fully adjusted model (Model 3): Age: continuous, in years; Sex: male or female; Educational level: categorized as high school and above, middle school, primary school, or no formal education; Residence: rural or urban; Marital status: married or otherwise (including divorced, widowed, separated, or never married); Smoking status: current smoker (yes/no), defined as having smoked more than 100 cigarettes in life and currently smoking; Drinking status: current alcohol user (yes/no), defined as consuming alcoholic beverages at least once per month in the past year; Systolic blood pressure (SBP): continuous, in mmHg; Diastolic blood pressure (DBP): continuous, in mmHg; Serum creatinine (Scr): continuous, in mg/dL; Blood urea nitrogen (BUN): continuous, in mg/dL; Low-density lipoprotein cholesterol (LDL-C): continuous, in mg/dL; High-density lipoprotein cholesterol (HDL-C): continuous, in mg/dL; Triglycerides (TG): continuous, in mg/dL (log-transformed as appropriate); Cancer history: yes/no, based on self-reported physician diagnosis of cancer or malignant tumor (excluding minor skin cancers).

Multiple imputation

The imputation model included all covariates used in the fully adjusted analysis: age, sex, educational level, marital status, residence, smoking status, drinking status, systolic and diastolic blood pressure, serum creatinine, blood urea nitrogen, LDL-C, HDL-C, TG, and cancer history. Five complete datasets were generated using the mice package in R.

Winsorization

To reduce the influence of outliers, TyG and ABSI indices were Winsorized at the 1st and 99th percentiles, i.e., values below the 1st percentile were set to the 1st percentile value, and values above the 99th percentile were set to the 99th percentile value.

Proportional hazards assumption test details

Full results of Schoenfeld residual tests, including all individual covariates, are presented in Supplementary Table S11.

Table S1. Characteristics of the study population according to TYG-ABSI at Wave 3

**Characteristic**	**TotalN = 5334**	**Q1 N = 1334**	**Q2 N = 1333**	**Q3 N = 1333**	**Q4 N = 1334**	**p-value**
Age, years (Mean ± SD)	62.24±8.70	61.73 ± 8.72	61.68 ± 8.59	61.79 ± 8.45	63.77 ± 8.84	<0.001
Sex, n (%)						<0.001
Female	2950(55.31%)	579 (43.40%)	701 (52.59%)	803 (60.24%)	867 (64.99%)	
Male	2384(44.69%)	755 (56.60%)	632 (47.41%)	530 (39.76%)	467 (35.01%)	
Education, n (%)						0.55
High school or above	479(8.98%)	124 (9.30%)	130 (9.75%)	122 (9.15%)	103 (7.72%)	
Middle school	1099(20.60%)	301 (22.56%)	268 (20.11%)	277 (20.78%)	253 (18.97%)	
Primary school	1220(22.87%)	294 (22.04%)	324 (24.31%)	326 (24.46%)	276 (20.69%)	
No formal education	2536(47.54%)	615 (46.10%)	611 (45.84%)	608 (45.61%)	702 (52.62%)	
Marriage, n (%)						0.008
Married	4621(86.63%)	1168 (87.56%)	1159 (86.95%)	1174 (88.07%)	1120 (83.96%)	
Other	713(13.37%)	166 (12.44%)	174 (13.05%)	159 (11.93%)	214 (16.04%)	
Residence, n (%)						<0.001
Rural	3610(67.68%)	984 (73.76%)	928 (69.62%)	889 (66.69%)	809 (60.65%)	
Urban	1724(32.32%)	350 (26.24%)	405 (30.38%)	444 (33.31%)	525 (39.35%)	
Hypertension, n (%)	1732(32.47%)	561 (42.37%)	639 (48.19%)	719 (54.31%)	834 (63.09%)	<0.001
Diabetes, n (%)	366(6.86%)	87 (6.52%)	127 (9.53%)	173 (12.98%)	389 (29.16%)	0.44
Heart disease, n (%)	693(12.99%)	166 (12.44%)	181 (13.58%)	185 (13.88%)	161 (12.07%)	<0.001

Characteristic	TotalN = 5334	Q1 N = 1334	Q2 N = 1333	Q3 N = 1333	Q4 N = 1334	p-value
Current smoker, n (%)	1398(26.21%)	442 (33.13%)	374 (28.10%)	290 (21.77%)	291 (21.81%)	<0.001
Current Drinker , n (%)	1778(33.33%)	892 (66.87%)	957 (71.90%)	1042 (78.23%)	1043 (78.19%)	<0.001
BMI, mmHg (Mean ± SD)	23.77±3.62	22.61 ± 3.52	23.40 ± 3.49	24.36 ± 3.52	24.71 ± 3.55	<0.001
SBP, mmHg (Mean ± SD)	127.75±19.92	124.06 ± 19.58	125.99 ± 19.49	128.73 ± 19.58	132.09 ± 20.12	<0.001
DBP, Mean (SD)	75.01±11.73	73.32 ± 11.18	74.48 ± 12.23	75.60 ± 11.22	76.66 ± 11.95	<0.001
HDL, mg/dL (Mean ± SD)	51.72±11.87	56.26 ± 13.30	53.20 ± 11.38	50.48 ± 10.83	46.94 ± 9.66	<0.001
LDL, mg/dL (Mean ± SD)	103.33±28.96	98.00 ± 26.75	104.74 ± 27.78	107.07 ± 29.05	103.51 ± 31.32	<0.001
TG, mg/dL (Mean ± SD)	141.87±89.56	83.57 ± 40.61	104.97 ± 38.18	144.03 ± 52.18	234.90 ± 113.29	<0.001
Uric acid, mg/dL (Mean ± SD)	4.88±1.39	4.64 ± 1.30	4.75 ± 1.33	4.97 ± 1.42	5.17 ± 1.43	<0.001
HbA1c, (Mean ± SD)	5.96±0.94	5.73 ± 0.49	5.79 ± 0.58	5.92 ± 0.77	6.39 ± 1.45	<0.001
Stroke, n (%)	96(1.80%)	24 (1.80%)	20 (1.50%)	32 (2.40%)	20 (1.50%)	0.25
CMM, n (%)	424(7.95%)	65(4.87%)	76(5.70%)	119(8.93%)	164(12.29%)	<0.001

BMI: body mass index; SBP: Systolic blood pressure; DBP:diastolic blood pressure; HbA1c: glycated haemoglobin; HDL-C: high-density lipoprotein cholesterol; LDL-C: low-density lipoprotein cholesterol; SD: standard deviation; TG: triglyceride; CMM: cardiometabolic multimorbidity.

Table S2. Cox regression analysis for the association between TyG-ABSI and CMM (using the original data analysis)

	Model 1		Model 2		Model 3	
	**HR (95%CI)**	**P value**	**HR (95%CI)**	**P value**	**HR (95%CI)**	**P value**
**TyG-ABSI (per 1 SD)**	1.51 (1.34, 1.70)	<0.001	1.43 (1.26, 1.61)	<0.001	1.37 (1.16, 1.62)	<0.001
1	1(Ref)		1(Ref)		1(Ref)	
2	1.17 (0.84, 1.63)	0.35	1.14 (0.82, 1.59)	0.44	1.10 (0.78, 1.54)	0.59
3	1.85 (1.37, 2.50)	<0.001	1.76 (1.29, 2.38)	<0.001	1.58 (1.14, 2.18)	0.01
4	2.59 (1.94,	<0.001	2.32 (1.73,	<0.001	2.08 (1.45,	<0.001

	Model 1		Model 2		Model 3	
	HR (95%CI)	P value	HR (95%CI)	P value	HR (95%CI)	P value
	3.45)		3.11)		2.98)	
Trend.test		<0.001		<0.001		<0.001
Cumulative TyG-ABSI **(per 1 SD)**	1.53 (1.38, 1.69)	<0.001	1.45 (1.31, 1.61)	<0.001	1.41 (1.23, 1.62)	<0.001
1	1(Ref)		1(Ref)		1(Ref)	
2	1.46 (1.04, 2.04)	0.03	1.40 (1.00, 1.97)	0.05	1.36 (0.96, 1.92)	0.08
3	1.88 (1.36, 2.59)	<0.001	1.76 (1.27, 2.43)	<0.001	1.63 (1.16, 2.29)	0.01
4	3.21 (2.38, 4.33)	<0.001	2.85 (2.10, 3.87)	<0.001	2.56 (1.79, 3.65)	<0.001
Trend.test		<0.001		<0.001		<0.001

Model 1 was unadjusted

Model 2 adjusted for age, sex

Model 3 adjusted for age, sex, history of cancer, marital status, smoking status, drinking status, educational level, residence, BUN, Scr, TG, HDL-C, LDL-C, SBP and DBP

Ref: reference; CI: confidence interval; HR: hazard ratio;

Table S3 Sensitivity analysis of the TyG-ABSI on the risks of developing CMM in participants without diabetes, heart disease or stroke(N=3720)

	Model 1		Model 2		Model 3	
	HR (95%CI)	P value	HR (95%CI)	P value	HR (95%CI)	P value
**TyG-ABSI (per 1 SD)**	1.45 (1.14, 1.86)	<0.001	1.43 (1.11, 1.83)	<0.001	1.29 (0.95, 1.75)	0.11
1	1(Ref)		1(Ref)		1(Ref)	
2	1.33 (0.73, 2.44)	0.35	1.35 (0.74, 2.46)	0.34	1.32 (0.70, 2.47) 0.39	0.59
3	2.44 (1.42, 4.21)	<0.001	2.49 (1.44, 4.32)	<0.001	2.16 (1.18, 3.98)	0.01
4	2.22 (1.25, 3.94)	0.01	2.17 (1.21, 3.89)	0.01	1.70 (0.80, 3.62)	0.17
Trend.test		<0.001		<0.001		0.05
Cumulative TyG-ABSI **(per 1 SD)**	1.41 (1.14, 1.75)	<0.001	1.38 (1.11, 1.71)	<0.001	1.25 (0.95, 1.64)	0.11
1	1(Ref)		1(Ref)		1(Ref)	
2	2.10 (1.13, 3.90)	0.02	2.07 (1.11, 3.85)	0.02	2.13 (1.11, 4.11)	0.02
3	2.67 (1.46, 4.88)	<0.001	2.63 (1.43, 4.82)	<0.001	2.46 (1.26, 4.81)	0.01
4	3.22 (1.75,	<0.001	3.06 (1.65,	<0.001	2.67 (1.27, 5.63)	0.01

	Model 1		Model 2		Model 3	
	HR (95%CI)	P value	HR (95%CI)	P value	HR (95%CI)	P value
	5.91)		5.68)			
Trend.test		<0.001		<0.001		0.01

Model 1 was unadjusted

Model 2 adjusted for age, gender

Model 3 adjusted for age, gender, smoking status, Serum creatinine , marital status, educational level， residence, drinking status, history of cancer, TG, SBP, DBP, blood urea nitrogen, HDL-C and LDL-C

Ref: reference; CI: confidence interval; HR: hazard ratio;

Table S4 Sensitivity analysis of the TyG-ABSI on the risks of developing CMM in participants without diabetes, heart disease, stroke and cancer (N=3679)

	Model 1		Model 2		Model 3	
	HR (95%CI)	P value	HR (95%CI)	P value	HR (95%CI)	P value
**TyG-ABSI (per 1 SD)**	1.45 (1.14, 1.86)	<0.001	1.42 (1.11, 1.82)	0.01	1.29 (0.95, 1.75) 0.11	0.11
1	1(Ref)		1(Ref)		1(Ref)	
2	1.32 (0.72, 2.41)	0.36	1.33 (0.73, 2.44)	0.35	1.32 (0.70, 2.47)	0.39
3	2.44 (1.42, 4.21)	<0.001	2.49 (1.44, 4.31)	<0.001	2.16 (1.18, 3.98)	0.01
4	2.20 (1.24, 3.91)	0.01	2.15 (1.19, 3.85)	0.01	1.70 (0.80, 3.62)	0.17
Trend.test		<0.001		<0.001		0.05
Cumulative TyG-ABSI **(per 1 SD)**	1.41 (1.14, 1.75)	<0.001	1.37 (1.11, 1.70)	<0.001	1.25 (0.95, 1.64)	0.11
1	1(Ref)		1(Ref)		1(Ref)	
2	2.09 (1.12, 3.88)	0.02	2.06 (1.11, 3.83)	0.02	2.13 (1.11, 4.11)	0.02
3	2.65 (1.45, 4.86)	<0.001	2.61 (1.42, 4.80)	<0.001	2.46 (1.26, 4.81)	0.01
4	3.21 (1.75, 5.88)	0.01	3.04 (1.64, 5.65)	<0.001	2.67 (1.27, 5.63)	0.01
Trend.test		<0.001		<0.001		0.01

Model 1 was unadjusted

Model 2 adjusted for age, sex

Model 3 adjusted for age, sex, history of cancer, marital status, smoking status, drinking

status, educational level, residence, BUN, Scr, TG, HDL-C, LDL-C, SBP and DBP

Ref: reference; CI: confidence interval; HR: hazard ratio;

Table S5: Comparison of characteristics between excluded and included populations.

Characteristic	Excluded (N=15,763)	Included (N=5,334)	Standardized Difference	P-value
Age, years	58.54 ± 11.21	62.24 ± 8.70	0.37	<0.001
Female, %	51.27%	55.31%	0.08	<0.001
Rural residence, %	56.62%	67.68%	0.23	<0.001
No formal education, %	38.43%	47.54%	0.24	<0.001
diabetes, %	18.28%	14.55%	0.1	<0.001
stroke, %	4.96%	1.80%	0.18	<0.001
heart disease, %	20.41%	12.99%	0.2	<0.001
SBP, mmHg	126.52 ± 20.17	127.71 ± 19.92	0.06	<0.001
HDL-C, mg/dL	50.80 ± 11.27	51.72 ± 11.87	0.08	<0.001

Table S6. Threshold effect analysis of TyG-ABSI on CMM risk in females

Inflection point of cumulative TyG-ABSI	Total/Event	HR (95% CI)	P value
< 1.86	222/12	0.31 (0.06,1.62)	0.165
≥1.86	2728/258	7.13 (3.19,15.91)	<0.001
Log-likelihood ratio		0.007	
**Inflection point of TyG-ABSI**			
< 0.61	154/5	0.46 (0.02, 9.01)	0.61
≥0.61	2796/265	*2.43 (1.07, 7.32)	0.032
Log-likelihood ratio		0.01	

adjusted for Model 2 adjusted for age, sex

Model 3 adjusted for age, sex, history of cancer, marital status, smoking status, drinking status, educational level, residence, BUN, Scr, TG, HDL-C, LDL-C, SBP and DBP

*In the female subgroup, the reference group (TyG-ABSI < 0.61) had a small number of events (<10). To avoid sparse-data bias, the Firth penalized likelihood method was used for stimation.

Table S7: Comparison of the predictive performance for CMM

Outcomes	IDI (95%CI)	*P* value	NRI (95%CI)	*P* value
CMM				
Basic model	–	–	–	–
Basic model + ABSI	0.018 (0.003, 0.033)	0.032	0.0624 (0.0024, 0.1223)	0.04
Basic model + TyG	0.020 (0.006, 0.034)	0.008	0.0714 (0.0230, 0.1198)	0.004
Basic model + TyG-ABSI	0.022 (0.006, 0.038)	0.007	0.0760 (0.0239, 0.1281)	0.004
Basic model + cumulative TyG-ABSI	0.032 (0.015, 0.049)	<0.001	0.1012 (0.0452, 0.1572)	<0.001

The basic model included age, sex, history of cancer, marital status, smoking status, drinking status, educational level, residence, BUN, Scr, TG, HDL-C, LDL-C, SBP and DBP

Table S8. Associations of single-point and cumulative TyG-ABSI with incident CMM and E-value analysis.

Exposure	HR (95%CI)	E-value	Upper limit of 95%CI
TyG-ABSI, per 1-SD increase	1.33 (1.13, 1.56)	1.88	1.49
Cumulative TyG-ABSI, per 1-SD increase	1.39 (1.21, 1.59)	2.01	1.67

Table S9 Sensitivity analysis of the TyG-ABSI on the risks of developing CMM

	Model 1		Model 2		Model 3	
	HR (95%CI)	P value	HR (95%CI)	P value	HR (95%CI)	P value
TyG-ABSI (per 1 SD)	1.45 (1.14, 1.86)	<0.001	1.42 (1.11, 1.82)	0.01	1.34 (1.18, 1.53) 0.11	0.11
1	1(Ref)		1(Ref)		1(Ref)	
2	1.32 (0.72, 2.41)	0.36	1.33 (0.73, 2.44)	0.35	1.09 (0.78, 1.53)	0.61
3	2.44 (1.42, 4.21)	<0.001	2.49 (1.44, 4.31)	<0.001	1.55 (1.12, 2.13)	0.01
4	2.20 (1.24, 3.91)	0.01	2.15 (1.19, 3.85)	0.01	1.97 (1.44, 2.71)	0.17

	Model 1		Model 2		Model 3	
	**HR (95%CI)**	**P value**	**HR (95%CI)**	**P value**	**HR (95%CI)**	**P value**
Trend.test		<0.001		<0.001		<0.001
Cumulative TyG-ABSI **(per 1 SD)**	1.41 (1.14, 1.75)	<0.001	1.37 (1.11, 1.70)	<0.001	1.36 (1.22, 1.53) <0.001	0.11
1	1(Ref)		1(Ref)		1(Ref)	
2	2.09 (1.12, 3.88)	0.02	2.06 (1.11, 3.83)	0.02	1.35 (0.96, 1.91)	0.09
3	2.65 (1.45, 4.86)	<0.001	2.61 (1.42, 4.80)	<0.001	1.59 (1.14, 2.23)	0.01
4	3.21 (1.75, 5.88)	0.01	3.04 (1.64, 5.65)	<0.001	2.43 (1.76, 3.37)	<0.001
Trend.test		<0.001		<0.001		0.01

Model 1 was unadjusted

Model 2 adjusted for age, sex

Model 3 adjusted for age, sex, history of cancer, marital status, smoking status, drinking status, educational level, residence, BUN, Scr, TG, HDL-C, LDL-C, SBP and DBP

Ref: reference; CI: confidence interval; HR: hazard ratio;

Table S10.1 Covariance analysis of cumulative TyG-ABSI and other variables based on model 3

Variable	GVIF	Df	GVIF^(1/(2×Df))
cumulative TyG-ABSI	1.777	1	1.333
marital status	1.144	1	1.07
Residenc	1.044	1	1.022
history of cancer	1.007	1	1.004
SBP	2.11	1	1.453
DBP	1.994	1	1.412
blood urea nitrogen	1.145	1	1.07
Serum creatinine	1.177	1	1.085
TG	1.83	1	1.353

HDL-C	1.287	1	1.134
LDL-C	1.108	1	1.052
age	1.604	1	1.267
educational level	1.241	3	1.037
drinking status	1.132	1	1.064
smoking status	1.107	1	1.052
gender	1.178	1	1.085

SBP, systolic blood pressure; DBP, diastolic blood pressure; HDL-C, high-density lipoprotein cholesterol; LDL-C, low-density lipoprotein cholesterol; TG, Triglyceride

Table S10.2 Covariance analysis of TyG-ABSI and other variables based on model 3

Variable	GVIF	Df	GVIF^(1/(2×Df))
tyg-absi	1.89	1	1.375
marital status	1.144	1	1.07
Residenc	1.048	1	1.024
history of cancer	1.007	1	1.003
SBP	2.112	1	1.453
DBP	2.006	1	1.416
blood urea nitrogen	1.141	1	1.068
Serum creatinine	1.178	1	1.085
TG	1.98	1	1.407
HDL	1.285	1	1.133
LDL	1.108	1	1.053

age	1.581	1	1.257
educational level	1.241	3	1.037
drinking status	1.133	1	1.065
smoking status	1.106	1	1.051
gender	2.911	1	1.706

SBP, systolic blood pressure; DBP, diastolic blood pressure; HDL-C, high-density lipoprotein cholesterol; LDL-C, low-density lipoprotein cholesterol; TG, Triglyceride

Table S11 Results of Schoenfeld residual-based tests for proportional hazards assumption in cumulative and single-point TyG-ABSI models

	χ^2	**df**	**p**
cumulative TyG-ABSI	0.235	1	0.628
age	2.24	1	0.134
sex	1.87	1	0.172
educational level	2.61	3	0.455
Residenc	1.08	1	0.299
marital status	0.228	1	0.633
smoking status	2.01	1	0.156
drinking status	0.000074	1	0.993
SBP	2.35	1	0.126
DBP	0.311	1	0.577
blood urea nitrogen	0.925	1	0.336
Serum creatinine	0.016	1	0.899
LDL	4.55	1	0.033
HDL	1.74	1	0.187
tg	1.58	1	0.208
history of cancer	0.005	1	0.943
global	24.6	18	0.135

SBP, systolic blood pressure; DBP, diastolic blood pressure; HDL-C, high-density lipoprotein cholesterol; LDL-C, low-density lipoprotein cholesterol; TG, Triglyceride

	χ^2	df	p
single-point TyG-ABSI	0.174	1	0.677
age	2.19	1	0.139
sex	1.9	1	0.168
educational level	2.58	3	0.462
Residenc	1.1	1	0.294
marital status	0.228	1	0.633
smoking status	2.02	1	0.155
drinking status	0.00003	1	0.996
SBP	2.36	1	0.124
DBP	0.307	1	0.58
blood urea nitrogen	0.958	1	0.328
Serum creatinine	0.02	1	0.888
LDL	4.62	1	0.032
HDL	1.81	1	0.179
tg	1.65	1	0.2
history of cancer	0.004	1	0.952
global	25.1	18	0.122

SBP, systolic blood pressure; DBP, diastolic blood pressure; HDL-C, high-density lipoprotein cholesterol; LDL-C, low-density lipoprotein cholesterol; TG, Triglyceride
